# Effects of Paid Leave Policies on Working Caregivers' Labor Supply

**DOI:** 10.1093/geroni/igaa057.099

**Published:** 2020-12-16

**Authors:** Soohyun Kim

**Affiliations:** Columbia University, New York, New York, United States

## Abstract

Approximately two in three caregivers are in the labor force. However, paid family leave is the only policy support for working caregivers to date, which helps to balance their work and care responsibilities. I analyze the data from the 1998-2014 Health and Retirement Study to examine 1) how paid leave policies affect labor market outcomes for workers in need of caring for a spouse/partner or an older parent/-in-law and 2) how the effects differ by gender. Paid leave policy is distinguished between employer-provided leave and state government-provided leave. Using the first-difference approach, I compare the short-term and long-term changes in the extensive and intensive margins of labor with and without access to paid leave policies when a health deterioration of the older family member occurs. My preliminary results show that, without paid leave policy, the health event of a spouse/partner or an older parent/-in-law affects women’s labor supply but not men’s. Paid leave provided by an employer increases the labor supply for both women and men, with the more noticeable long-term effects for men. State paid family leave increases women’s wage and salary both in the short run and in the long run. My findings underline the importance of paid leave policy for retaining the workers in need of providing care for a family member, particularly for women.

